# Production of Destruxins from *Metarhizium* spp. Fungi in Artificial Medium and in Endophytically Colonized Cowpea Plants

**DOI:** 10.1371/journal.pone.0104946

**Published:** 2014-08-15

**Authors:** Patrícia S. Golo, Dale R. Gardner, Michelle M. Grilley, Jon Y. Takemoto, Stuart B. Krasnoff, Marcus S. Pires, Éverton K. K. Fernandes, Vânia R. E. P. Bittencourt, Donald W. Roberts

**Affiliations:** 1 Departamento de Parasitologia Animal, Instituto de Veterinária, Universidade Federal Rural do Rio de Janeiro, Seropédica, RJ, Brazil; 2 Department of Biology, Utah State University, Logan, Utah, United States of America; 3 USDA, ARS, Poisonous Plants Research Laboratory, Logan, Utah, United States of America; 4 Biological Integrated Pest Management Research Unit, Robert W. Holley Center for Agriculture and Health, USDA-ARS, Ithaca, New York, United States of America; 5 Instituto de Patologia Tropical e Saúde Pública, Universidade Federal de Goiás, Goiânia, GO, Brazil; Centro de Investigación y de Estudios Avanzados, Mexico

## Abstract

Destruxins (DTXs) are cyclic depsipeptides produced by many *Metarhizium* isolates that have long been assumed to contribute to virulence of these entomopathogenic fungi. We evaluated the virulence of 20 *Metarhizium* isolates against insect larvae and measured the concentration of DTXs A, B, and E produced by these same isolates in submerged (shaken) cultures. Eight of the isolates (ARSEF 324, 724, 760, 1448, 1882, 1883, 3479, and 3918) did not produce DTXs A, B, or E during the five days of submerged culture. DTXs were first detected in culture medium at 2–3 days in submerged culture. *Galleria mellonella* and *Tenebrio molitor* showed considerable variation in their susceptibility to the *Metarhizium* isolates. The concentration of DTXs produced *in vitro* did not correlate with percent or speed of insect kill. We established endophytic associations of *M. robertsii* and *M. acridum* isolates in *Vigna unguiculata* (cowpeas) and *Cucumis sativus* (cucumber) plants. DTXs were detected in cowpeas colonized by *M. robertsii* ARSEF 2575 12 days after fungal inoculation, but DTXs were not detected in cucumber. This is the first instance of DTXs detected in plants endophytically colonized by *M. robertsii*. This finding has implications for new approaches to fungus-based biological control of pest arthropods.

## Introduction

Despite concerns with negative impacts of chemical insecticides on human health, the use of these chemicals remains high. Consequently, the demand for alternatives is increased. Biological control of arthropod pests using entomopathogenic fungi is one promising alternative [1], [2], [3]. Entomopathogenic fungi from the genus *Metarhizium* are some of the most frequently studied biological control agents for use against insects and ticks [2], [3], [4].

*Metarhizium* spp. produce a wide array of small molecules including destruxins (DTXs), cyclic depsipeptides which are produced as well as by some other fungi, both insect (*Aschersonia*) and plant pathogens (*Alternaria*, *Trichothecium*) [5]. The effects of DTXs on insects include: tetanic paralysis [6], [7], inhibition of DNA and RNA synthesis in insect cell lines [8], inhibition of Malpighian tubule fluid secretion [9], blocking H^+^ ATPase activity [10], and suppression of insect defense responses [11], [12], [13], [14], [15]. DTXs also have antifeedant and repellent properties [16], [17]. The insecticidal potential of these toxins has been confirmed in numerous reports of acute toxicity [5]. Despite demonstrated insecticidal activity of DTX, Donzelli et al. [18] showed that a *Metarhizium robertsii* mutant with disrupted DTX synthetases was as virulent as the wild type strain when fungus conidia were topically applied to insect larvae. This supports the conclusions of a previous report that *Metarhizium* spp. isolates could be pathogenic for insects whether they had the ability to produce *in vitro* DTXs or not [19]. Although these compounds have been detected in moribund, infected hosts [20], [21], DTXs reportedly have little or no impact on virulence as measured in whole-insect bioassays [18], [19].

DTXs also have negative effects on insect behavior, for example inducing phagodepression and repellence [16], [17]. *Metarhizium robertsii* (ARSEF 2575) is plant-rhizosphere competent and has endophytic capability [22], [23], [24], [25]; accordingly, if DTXs produced inside *Metarhizium*-colonized plants induced antifeedant effects on arthropod pests of those plants, then the presence of DTXs *in planta* may afford enhanced levels of *Metarhizium*-associated biological control of these pest arthropods.

We report here a survey of virulence of 20 *Metarhizium* isolates against insect larvae, and the concentration of DTXs A, B, and E produced by these same isolates *in vitro* (submerged shake cultures). We then analyzed plants endophytically colonized by a high-DTX producing *M. robertsii* isolate and a low- or non-DTX producing *M. acridum* isolate [26], [27] to search for DTXs in colonized plants.

## Material and Methods

### Fungal isolates

Twenty *Metarhizium* spp. isolates were used in the present study: 18 isolates from different regions of Brazil, one from the USA and one from Australia (Table 1). Fungal isolates were obtained from the Agriculture Research Service Collection of Entomopathogenic Fungal Cultures (ARSEF) (USDA-US Plant, Soil and Nutrition Laboratory, Ithaca, NY, USA). Stock cultures were grown on PDAY (potato dextrose agar plus 0.01% yeast extract) at 27°C for 14 days and then held at 4°C. Conidia for all experiments were produced on PDAY 60×60 mm Petri plates and incubated at 27°C for 14 days. Conidia were harvest by scraping using a bacterial loop and suspended in 0.01% Tween 80 in 15-mL centrifuge tubes (Modified polystyrene, Corning inc., Corning, NY, USA) and vigorously agitated (vortexed). Conidial viability was measured by placing a 50 µL drop of fungal suspension on a PDAY plate and germination was observed by compound microscope (400×) after 24 hours at 28°C.

**Table 1:** *Metarhizium* spp. isolates used in this study, including their hosts and origins (state and country).

Fungal Isolate	Host/Substrate	Origin	Species
ARSEF 324	*Austracris guttulosa* (Orthoptera: Acrididae)	QLD, Australia	*Metarhizium acridum*
ARSEF 552	Lepidoptera	MG, Brazil	*Metarhizium pingshaense*
ARSEF 724	*Cerotoma arcuata* (Coleoptera:Chrysomelidae)	GO, Brazil	*Metarhizium robertsii*
ARSEF 729	*Deois flavopicta* (Homoptera: Cercopidae)	GO, Brazil	*Metarhizium anisopliae sensu lato* (s.l.)
ARSEF 759	*Deois flavopicta* (Homoptera: Cercopidae)	GO, Brazil	*Metarhizium anisopliae* s.l.
ARSEF 760	*Cerotoma arcuata* (Coleoptera: Chrysomelidae)	GO, Brazil	*Metarhizium anisopliae* s.l.
ARSEF 782	*Deois flavopicta* (Homoptera: Cercopidae)	GO, Brazil	*Metarhizium anisopliae* s.l.
ARSEF 929	*Chalcodermus aeneus* (Coleoptera: Curculionidae)	GO, Brazil	*Metarhizium anisopliae* s.l.
ARSEF 1448	*Scaptores castanea* (Hemiptera: Cydnidae)	GO, Brazil	*Metarhizium pingshaense*
ARSEF 1449	*Deois flavopicta* (Homoptera: Cercopidae)	PA, Brazil	*Metarhizium anisopliae* s.l.
ARSEF 1882	*Tibraca limbativentris* (Hemiptera: Pentatomidae)	GO, Brazil	*Metarhizium anisopliae* s.l.
ARSEF 1883	*Tibraca limbativentris* (Hemiptera: Pentatomidae)	GO, Brazil	*Metarhizium anisopliae sensu stricto*
ARSEF 1885	*Diabrotica* sp. (Coleoptera: Chrysomelidae)	GO, Brazil	*Metarhizium anisopliae* s.l.
ARSEF 2211	Soil	SP, Brazil	*Metarhizium anisopliae* s.l.
ARSEF 2521	*Deois* sp. (Homoptera: Cercopidae)	PR, Brazil	*Metarhizium anisopliae* s.l.
ARSEF 2575	*Curculio caryae* (Coleoptera: Curculionidae)	SC, USA	*Metarhizium robertsii*
ARSEF 3479	(Coleoptera: Scarabaeidae)	DF, Brazil	*Metarhizium anisopliae* s.l.
ARSEF 3641	Soil	GO, Brazil	*Metarhizium anisopliae* s.l.
ARSEF 3643	Soil	GO, Brazil	*Metarhizium anisopliae* s.l.
ARSEF 3918	Soil	PR, Brazil	*Metarhizium anisopliae* s.l.

* USDA-ARS Collection of Entomopathogenic Fungal Cultures, Ithaca, NY.

Identifications were provided September 2012 by curator of ARSEF* Richard Humber.

### *In vitro* production of DTXs and HPLC-UV analysis

For the analysis of *in vitro* DTXs production, fungal cultures were started with 1×10^6^ conidia/100 mL CZAPEK-DOX Broth (BD Difco) with bactopeptone (0.5%) and incubated in 250-mL flasks at room temperature (∼22°C) on a rotary shaker at 150 rpm for 1, 2, 3, 4, or 5 days. Control isolates were *M. robertsii* ARSEF 2575 (a high DTX producer) and *Metarhizium acridum* ARSEF 324 (a low or non DTX producer) [26], [27]. Production of DTX in the culture supernatants was determined by quantitative HPLC-UV analysis of the major components (DTXs A, B and E). All solvents used in the current study were HPLC grade. Cultures were separated into fungus mycelium and supernatant by centrifugation at 1000 × g for 20 minutes. Mycelia were harvested, dried at 80°C for 48 hours, and weighed to obtain the amount of mycelial production for each isolate. Extraction of DTXs from culture supernatants was accomplished by loading 5 mL aliquots onto C18-SPE cartridges (100 mg; Agilent Bond Elut #12102001) that were previously conditioned with 10 column volumes of methanol followed by a similar volume of ultra-pure water. The loaded cartridges were rinsed with 10 mL ultra pure water and then eluted with 2 mL methanol [18].

Just prior to analysis the methanol extracts were diluted 1∶1 with water and then 10-µL aliquots of extract were injected onto a reversed phase (RP) Betasil C18 column (100 mm×2.1 mm, Thermo Fisher) with a guard column of the identical phase. Elution was with a gradient of acetonitrile and water using a modular HPLC system (Shimadzu Corp., Kyoto, Japan). The linear gradient conditions using the solvents A (acetonitrile) and B (water) were: 0–10 min (25% A increased to 60% A); 10–13 min (isocratic 60% A); 13–15 min (60% A decreased to 25% A) at a flow rate of 0.3 mL min^−1^. Detection was by UV absorbance at 220 nm. After the run was complete, the column re-equilibration time was 5 min. DTXs A, B, and E were measured using standard curves for each compound. DTXs A, B, and E standards were purified using methods based on those of Krasnoff et al. [28] and standard solutions prepared at 1 mg/mL in methanol. Calibration standards were prepared by dilution of 20 µL of each standard stock solution into 0.940 mL of 50% methanol and then serial dilution to give standards at 20, 10, 5, 2.5, 1.25, 0.62 and 0.31 µg mL^−1^. Limit of detection (LOD) was estimated to be 0.10 µg/mL based on a S/N ratio of 3 for UV detection at 220 nm.

### Detection of DTXs in plants

#### (i) Fungal inoculation of plants

Seeds of cowpea (*V. unguiculata*) (organic seeds, Shangri-la Health Foods, Logan, UT, USA) and cucumber (*C. sativus*) (“Straight Eight” untreated organic seeds, Snow Seed, Salinas, CA, USA) were weighed individually and only those weighing between 0.2500 g and 0.2599 g for cowpeas, and 0.0240 g and 0.0249 g for cucumber were used. Seeds were surface sterilized by immersion in 95% ethanol for 2 minutes, rinsed in sterile deionized water followed by immersion in 30% hydrogen peroxide for 1 minute. Disinfected seeds were then rinsed 3 times in sterile deionized water [29]. These axenic seeds were kept overnight at 4°C to synchronize growth. After synchronization, seeds were immersed for 1 h in conidial suspensions (1×10^6^ conidia mL^−1^ 0.01% Tween 80) of ARSEF 2575 or ARSEF 324. Seeds were then individually set on sterile, moist filter paper in Petri plates and kept at 25°C for 12 days with a photoperiod of 16∶8 (L∶D) (white fluorescent tubes [30]). Sterile water was added as needed to keep the filter paper moist. Uninoculated seeds (no-fungus control) were immersed in sterile deionized water containing 0.01% Tween 80 [23]. After 12 days, presence or absence of *M. robertsii* or *M. acridum* in plants was confirmed by incubating surface sterilized leaves, stems and roots on artificial medium. Surface sterilization was by immersion for 2 minutes in 0.5% sodium hypochlorite, 2 minutes in 70% ethanol, rinsed in sterile deionized water 3 times and dried using sterile filter papers. The outer edges of the leaves were dissected and discarded [31]. The remaining parts were cut into pieces and cultured on PDAY medium supplemented with 0.05% chloramphenicol in a 60 mm Petri plate. Three plates from each treatment (ARSEF 2575 exposed, ARSEF 324 exposed, or not-infected plants) were incubated with 2 or 3 pieces of leaf, stem or root per plate. The plates were examined daily for 7 days. Fungi growing from plant tissues were isolated and characterized morphologically according to Tulloch [32].

#### (ii) Extraction and LC-MS/MS analysis

After 12 days of growth, 10 plants of each treatment (ARSEF 2575 exposed; ARSEF 324 exposed; and not exposed) were frozen in liquid nitrogen and ground with mortar and pestle to a powder. To verify the accuracy of the DTX detection method, pure DTX standards (A, B, and E, 16.5 µg each) were mixed (before liquid nitrogen freezing and homogenization) into ten additional not-fungus-exposed 12-day-old plants. Methanol (5 mL) was added to each plant powder, followed by 15 mL ultra pure water. Plant suspensions were clarified by filtration (Whatman N° 1). Extractions of filtrates (20 mL) were carried out with C18 SPE cartridges (as described before).

The concentrations of DTXs A, B, and E, were measured by liquid chromatography-mass spectrometry (LC–MS). The LC-MS system consisted of a Betasil C18 RP HPLC column (100×2.1 mm, Thermo Fisher), coupled to a Surveyor MS Pump Plus, a Surveyor Auto Sampler Plus and a PDA UV–vis absorbance detector in-line with an LCQ Advantage Max mass spectrometer and electrospray (esi) ionization source (Thermo Electron Corp, San Jose, CA, USA). Sample injection size was 5-µL. The gradient-elution steps were the same as those used for LC-UV analysis (see section 2.2). Pseudomolecular ions [M+H]^+^ of DTX A, B, and E were observed at *m/z* 578, 594 and 594 respectively with the following retention times: 7.05 min (DTX A), 9.12 min (DTX B) and 5.03 min (DTX E). DTXs A, B, and E concentrations were measured using a standard curve for each compound prepared by serial dilutions as previously described (section 2.2); but with the lowest standard at 0.15 µg mL^−1^. The limit of detection (LOD) with the LC-MS system was estimated to be 0.010 µg mL^−1^ based on a S/N ratio of 3.

### Effect of DTXs on plant dry weight

After DTXs extraction, plant powders were held for 48 hours at 80°C and their dry weights' determined. Dry weights of fungus-treated plant groups and the non-treated groups were analyzed by analysis of variance (ANOVA) followed by the Tukey test with a significance level of 5% (*P*≤0.05) [33].

### Insect virulence assays

Bioassays were performed with *G. mellonella* (waxworms) and *T. molitor* (mealworms). Conidia of each isolate (Table 1) were used to prepare fungal suspensions (section 2.1). Conidial concentrations were estimated by hemocytometer counts and adjusted to 1×10^7^ and 1×10^5^ conidia mL^−1^.

Commercially produced *G. mellonella* larvae (last instar; 239 mg average weight) and *T. molitor* larvae (at least ninth instar; 95 mg average weight) (Fluker Farms, Port Allen, LA, USA) were treated either with 1×10^7^ conidia mL^−1^ or 1×10^5^ conidia mL^−1^. Two groups of 8 last instar *G. mellonella* larvae and 2 groups of 10 *T. molitor* larvae were placed in 60×15 mm polystyrene Petri dishes lined with a 5.5 cm P4 filter paper (Fisherbrand, Porosity: Medium – Fine, Flow rate: Slow) moistened with 0.5 mL sterile distilled water. Each plate containing larvae was sprayed with 0.5 mL fungal suspension. Control plates were sprayed with 0.01% Tween 80 solution. Plates were incubated at 28°C and ≥80 RH. Insect mortality was assessed daily for 10 days. The bioassays were repeated 3 times.

Mean larval mortalities at 3 days with *G. mellonella* and 5 days with *T. molitor* were compared using the non-parametric Kruskal-Wallis test for statistical differences. Comparison between the mean mortalities was performed using Student-Newman–Keuls (SNK) test. Data analyses were conducted using BioEstat software, version 4.0. *P*-values less than 0.05 were considered to be significant [34].

## Results

Conidial viability (percent germination) of all suspensions used in *in vitro* DTX production, plant-seed inoculations, and insect bioassays was at least 98%.

### *In vitro* production of DTXs

**Figure 1:**
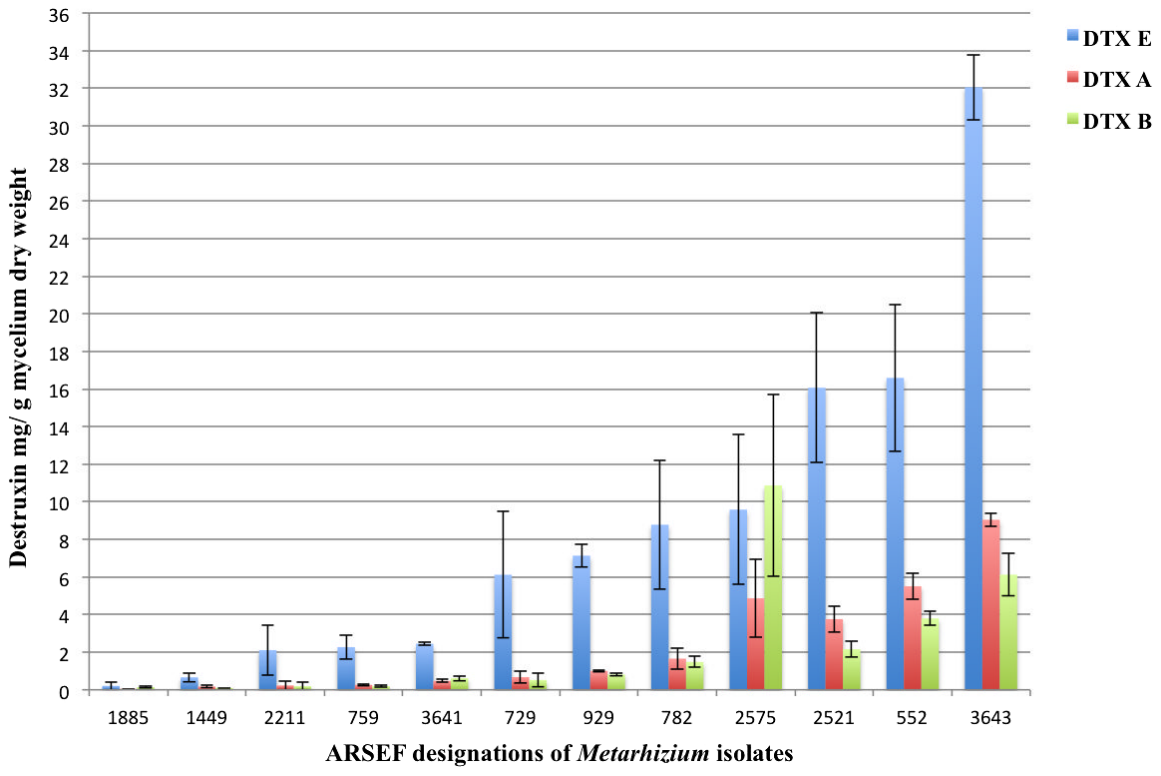
Destruxin (DTX) production by 12 *Metarhizium* spp. isolates *in vitro*. DTXs production is represented by mean values ± standard error after 5 days in submerged shaken cultures. Production of DTXs in supernatant of cultures was determined by quantitative HPLC analysis of the major components, viz., DTXs A, B and E. Cultures and assays were repeated 3 times.

Of the 20 *Metarhizium* spp. isolates examined in the current study, one (ARSEF 2575) was previously known to produce high levels of DTXs and one (ARSEF 324) to produce low levels of DTXs. In addition, in the present study, seven other isolates (ARSEF 724, 760, 1448, 1882, 1883, 3479, and 3918) did not produce DTXs *in vitro*. Among the DTXs producers (ARSEF 552, 729, 759, 782, 929, 1449, 1885, 2211, 2521, 3641, and 3643), production ranged from 0.31 mg DTX A/g dry weight (d.w.) of ARSEF 1885 mycelium to 32 mg DTX E/g d.w. of ARSEF 3643 mycelium, at 5 days after inoculation of conidial suspensions into liquid medium (Figure 1). Table S1 shows DTXs production *in vitro* represented by mg DTXs per L liquid media.

**Figure 2:**
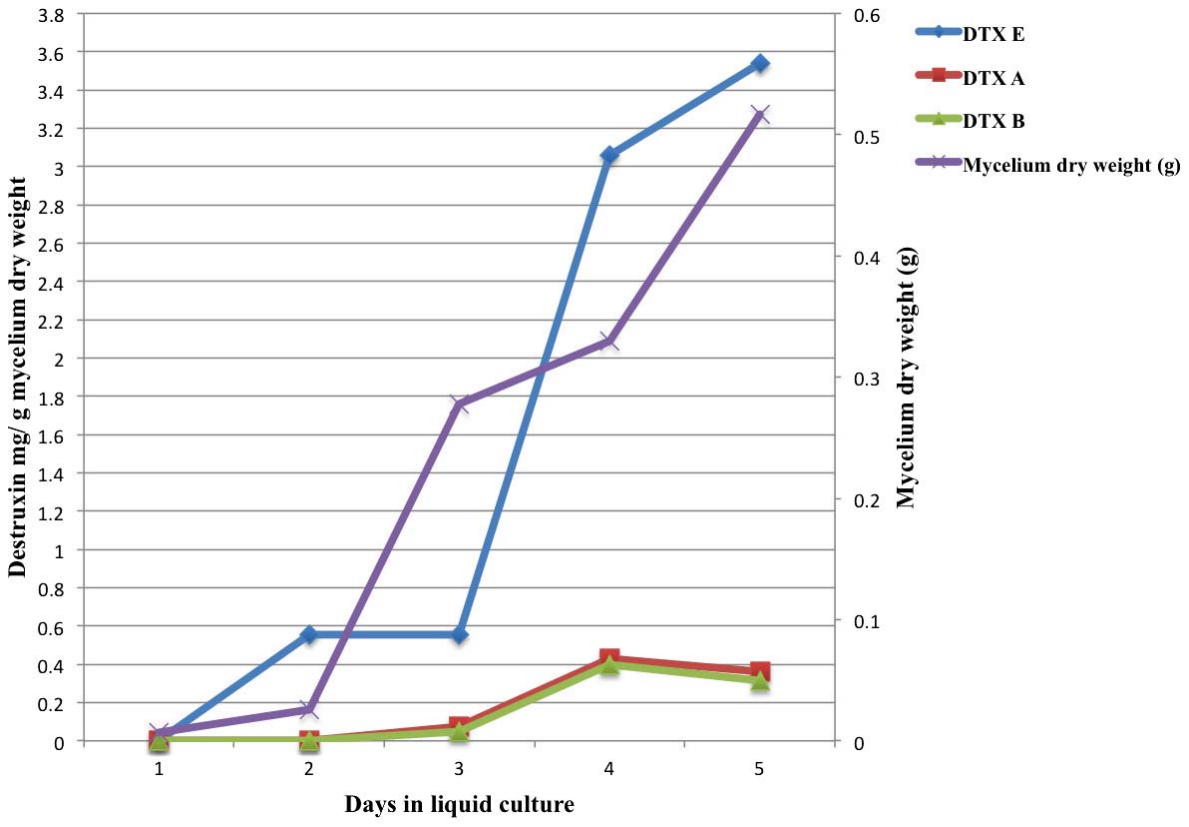
Time course of *in vitro* production of DTXs A, B, and E by *Metarhizium anisopliae* s.l. ARSEF 759. Destruxin concentrations in supernatants of submerged liquid cultures were determined by quantitative HPLC-UV analysis of the major components, viz., DTXs A, B and E. Values are expressed in mg DTXs per g dry weight mycelium.

**Figure 3:**
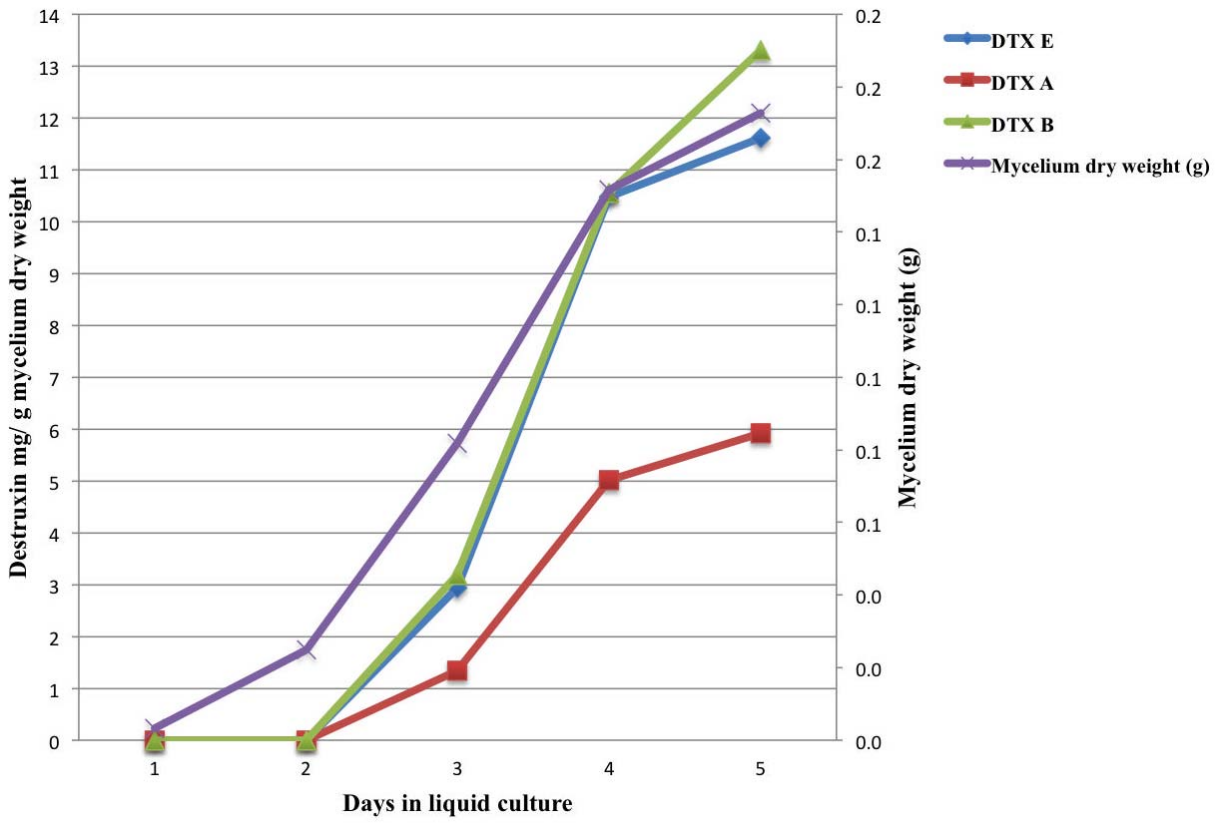
Time course of *in vitro* production of DTXs A, B, and E by *Metarhizium robertsii* ARSEF 2575. Destruxin concentrations in supernatant of submerged liquid cultures were determined by quantitative HPLC analysis of the major components, viz., DTXs A, B and E. Values are expressed in mg DTXs per g dry weight mycelium.

Generally, the earliest detection of DTXs in *in vitro* cultures was at day 3; the exception being ARSEF 759, which produced DTX E at day 2 (0.55 mg DTX E/g d.w. mycelium) (Figure 2). Two isolates (ARSEF 1885 and ARSEF 729) did not produce DTXs until 4 days in culture. The time course (from day 1 to day 5) of DTXs production by *M. anisopliae* s.l. (ARSEF 759) and *M. robertsii* (ARSEF 2575) (used as control isolate) is shown in Figures 2 and 3.

### Detection of *Metarhizium*-produced DTXs in plants

**Figure 4:**
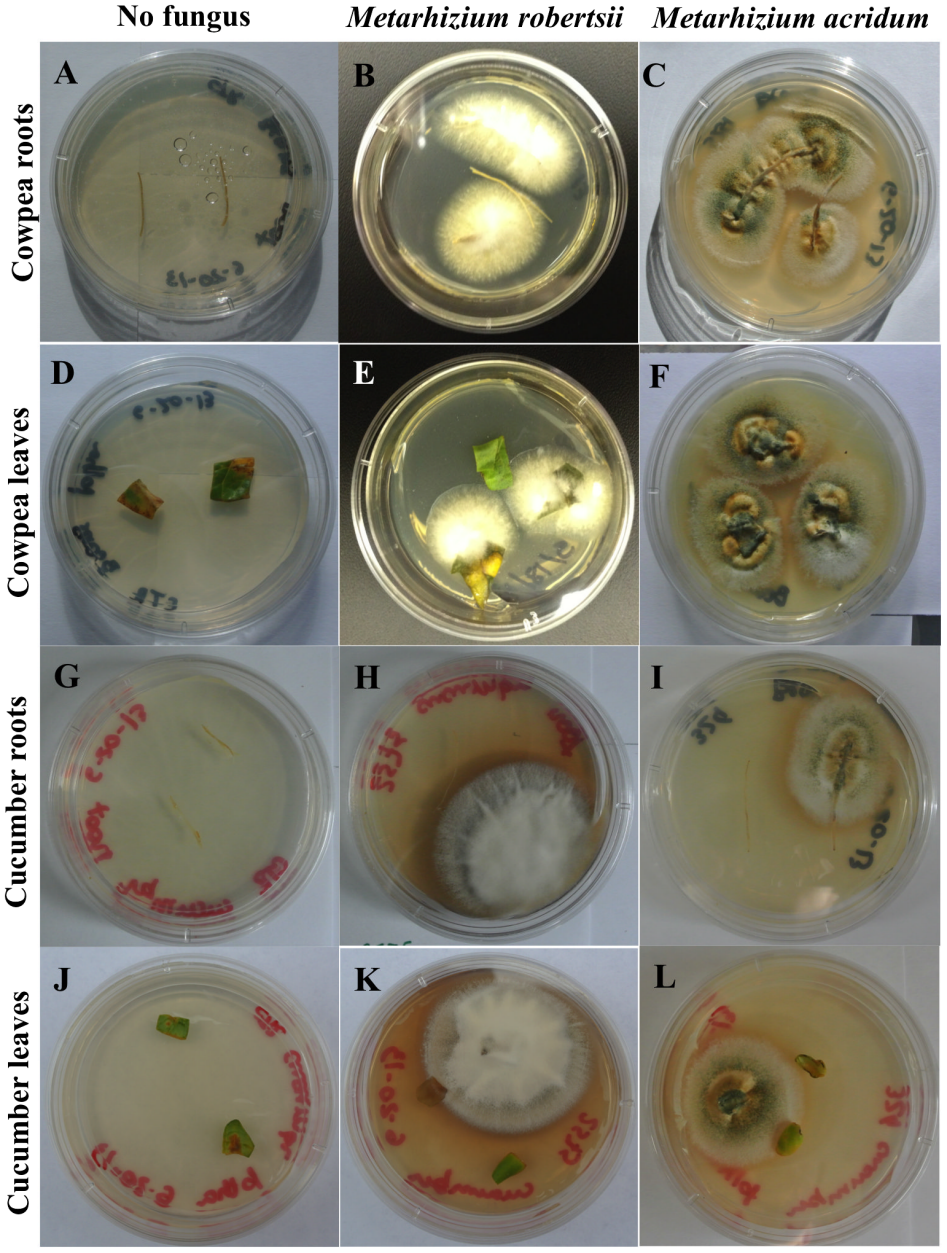
Re-isolation of *Metarhizium robertsii* or *M. acridum* after their endophytic colonization of cowpeas (*Vigna unguiculata*) and cucumber (*Cucumis sativus*). Control plants with no fungus inoculation (A, D, G, and J); *M. robertsii* growing from surface sterilized roots (B) and leaves (E) of cowpeas; *M. robertsii* growing from surface sterilized roots (H) and leaves (K) of cucumber. *M. acridum* growing from surface sterilized roots (C) and leaves (F) of cowpeas; and *M. acridum* growing from surface sterilized roots (I) and leaves (L) of cucumber. Note that the characteristic brownish-green conidia of *M. robertsii* were obscured by a layer of white mycelium, whereas the dark green conidia of *M. acridum* were more visible due to very little mycelial overlay.

Endophytic growth in 12-day-old cowpea (*V. unguiculata*) and cucumber (*C. sativus*) by *M. robertsii* and *M. acridum* was confirmed (Figure 4). In each case, the isolated fungus colonies presented the key morphological features consistent with *Metarhizium* isolates.

**Figure 5:**
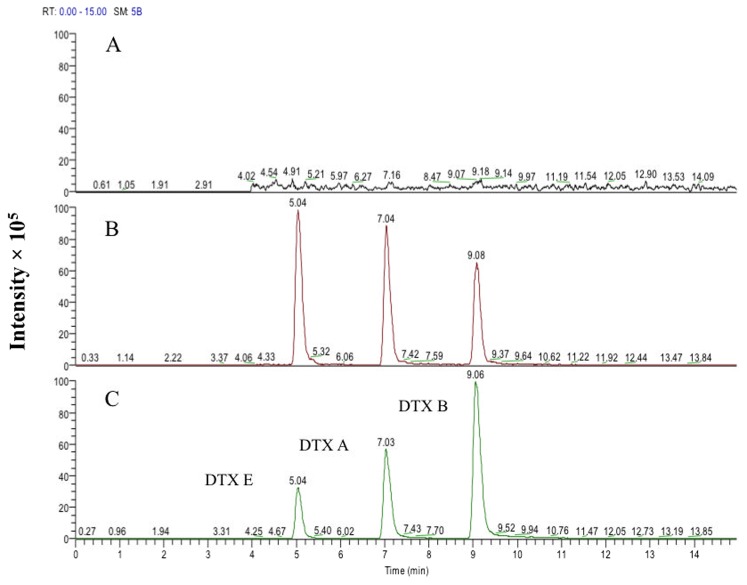
HPLC-MS analysis of cowpea extracts for destruxin (DTX) production. (A) Analysis of not colonized (free of fungus) plants (negative control); (B) plants endophytically colonized by *Metarhizium robertsii* ARSEF 2575; and (C) not-colonized plants spiked with DTX standards (positive control). The cowpea seeds, both fungus-inoculated and control (not colonized) were incubated on moist filter paper under optimal light (16L∶8D) and temperature (25°C) conditions for 12 days at which time the germlings had developed roots, stems, cotyledons and two true leaves. DTXs were extracted from entire plants using methanol 100% and SPE-C18 cartridges.

Detectable levels of DTXs A, B, and E were identified in combined roots, stems and leaves of cowpea plants cultured for 12 days after exposure of their seeds to *M. robertsii* conidia (Figure 5B). The concentrations of each compound followed by its respective standard error were: 5.73±0.29 µg DTX E/g d.w. cowpeas; 1.56±0.29 µg DTX A/g d.w. cowpeas; and 0.82±0.11 µg DTX B/g d.w. cowpeas. With cucumber, however, despite confirmation of *M. robertsii* endophytic colonization, no DTXs were detected in extracts of these plants. Also, no DTXs were detected in plants (cowpeas or cucumber) colonized by *M. acridum*, nor in control plants (not-infected plants). DTXs were detected in all positive controls (not-infected cowpea and cucumber plant tissues spiked with DTX standards) (Figure 5C).

### Effect of DTXs on dry weights of plants

No differences in total dry weights were noted between endophytic *Metarhizium*-colonized and not-colonized plants (*P*≥0.05). Similarly, for both plant species (*V. unguiculata* and *C. sativus*) there were no statistical differences between dry weights of plants endophytically colonized by a fungus that produces DTXs (*M. robertsii*) and plants colonized by a non-DTXs producer (*M. acridum*).

### Insect virulence assays

The virulence of 20 *Metarhizium* spp. isolates (Table 1) was surveyed using two different insect hosts: *G. mellonella* (waxworm) and *T. molitor* (mealworm). Natural mortality of untreated (control) *G. mellonella* larvae was always higher than with *T. molitor* larvae; e.g., waxworm control mortality reached 16.67% at day 5 while mealworm mortality was 1.6% at the same time. There were variations in the virulence of the isolates, and in the susceptibility of the different host species (Table 2). *T. molitor* larvae were less susceptible than *G. mellonella* larvae. For this reason, *T. molitor* bioassay data at day 5 after treatment were used for comparisons, and with *G. mellonella* day 3 data were used (Table 2). According to this evaluation system, several isolates (e.g., ARSEFs 724, 1448, 1885, 2575, 3641, and 3643) had similar levels of virulence for both species of insect.

**Table 2:** Mean mortality (%) ± standard error of *Tenebrio molitor* larvae 5 days after treatment, and *Galleria mellonella* 3 days after treatment.

	*Tenebrio molitor*		*Galleria mellonella*	
Fungal Isolates	Conidia concentration (mL^−1^)		Conidia concentration (mL^−1^)	
	1×10^7^	1×10^5^	1×10^7^	1×10^5^
ARSEF 3643	100.00±0.0 d	45.00±23.63 bce	100.00±0.0 f	14.58±8.33 a
ARSEF 1448	100.0±0.0 d	28.33±15.90 bcde	84.38±12.76 bdf	31.25±25.52 a
ARSEF 1885	96.67±1.7 cd	66.67±23.33 b	100.00±0.0 f	27.08±21.14 a
ARSEF 724	95.00±5.0 cd	45.00±22.55 bce	100.00±0.0 f	8.33±2.08 a
ARSEF 760	93.33±6.7 cd	13.33±7.26 ac	100.00±0.0 f	4.17±2.08 a
ARSEF 2575	93.33±6.7 cd	28.33±21.86 bc	100.00±0.0 f	20.83±20.83 a
ARSEF 1449	90.00±7.6 bcd	16.67±14.24 ac	97.92±2.08 df	8.33±5.51 a
ARSEF 782	88.33±9.3 bcd	16.67±7.26 bce	97.92±2.08 df	14.17±8.70 a
ARSEF 3641	86.67±8.8 bcde	33.33±20.28 bcde	100.00±0.0 f	53.13±38.27 a
ARSEF 2521	80.0±11.5 bcde	28.33±23.33 bce	68.75±31.25 bdefg	34.38±28.07 a
ARSEF 929	75.00±15.3 acd	16.67±14.24 ac	52.08±28.94 abd	20.83 12.67 a
ARSEF 759	66.67±20.3 abcd	3.33±3.33 ade	79.17±20.83 bcdf	16.67±9.08 a
ARSEF 552	63.33±11.7 abcd	0.00±0.0 a	89.58±10.42 bdf	0.00±0.0 a
ARSEF 2211	61.67±25.9 abd	5.00±2.89 ac	70.83±29.17 bdefg	25.00±18.75 a
ARSEF 729	46.67±14.8 abc	1.67±1.67 ade	64.58±26.60 bcdf	10.42±5.51 a
ARSEF 3918	40.00±30.6 abc	0.00±0.0 a	35.42±29.39 ab	53.13±38.27 a
ARSEF 1883	21.67±10.9 ab	0.00±0.0 a	33.33±18.52 ace	27.08±24.03 a
ARSEF 1882	16.67±14.2 ab	5.00±0.0 ac	35.42±18.52 ace	8.33±8.33 a
ARSEF 324	13.33±10.9 ae	0.00±0.0 a	31.25±15.73 acg	4.17±4.17 a
ARSEF 3479	5.00±5.0 a	1.67±1.67 ad	6.25±0.0 a	8.33±2.08 a
Control	1.67±1.7 a	1.67±1.7 ad	4.17±4.17 a	4.17±4.17 a

Bioassays were performed 3 times (using two replicates for each isolate) under controlled conditions (27°C), using new batches of larvae and conidia in each bioassay. Controls were treated with Tween 80 (0.01%) solution. Means followed by the same letter in a column do not differ statistically (*P* ≥ 0.05) (Kruskal-Wallis test followed by Student-Newman-Keuls).

With the highest concentration (10^7^ conidia mL^−1^), isolates ARSEF 724, 760, 1885, 2575, 3641, and 3643 caused 100% *G. mellonella* larval mortality at day 3 after treatment (Table 2). At day 5 after treatment with 10^7^ conidia mL^−1^, another 7 isolates (ARSEF 552, 729, 759, 782, 1448, 1449, and 2521) had already caused 100% *G. mellonella* mortality. In contrast, within the same 5 days, only 2 isolates (ARSEF 3643 and ARSEF 1448) caused 100% mortality of *T. molitor*; however, 4 other isolates (ARSEF 724, 760, 1885, and 2575) were sufficiently virulent to cause more than 90% mortality of *T. molitor* larvae at day 5 after treatment (Table 2).

ARSEF 552, 724, 729, 759, 782, 1449, 1882, 1885, 2521, 2575, 3641, 3643, 3479, and 3918 caused ≥ 50% *Galleria* larval mortality with the low-concentration treatment at day 5 after treatment. The most virulent isolates were ARSEF 1449 (91.67% larval mortality ±8.33 standard error), ARSEF 3643 (90.63% larval mortality ±9.38 se), and ARSEF 3643 (84.38% larval mortality ±15.63 se).

With *Tenebrio* at day 5, only ARSEF 1885 caused ≥50% mortality (66.67%±23.3 se) in the low-concentration treatment. ARSEF 3643 and ARSEF 724 were the second and third most virulent isolates with each causing 45% larval mortality (±23.6 se and 22.5 se, respectively) at day 5 after treatment with the low fungus concentration.

## Discussion

The present study investigated 20 *Metarhizium* spp. isolates as to their virulence against two insect species and their levels of DTXs production in artificial liquid medium. A wide variation in DTXs production *in vitro* was observed among the 13 isolates in the present study (Figure 1). Two *Metarhizium* isolates were used to analyze fungus-colonized cowpea and cucumber plants for DTXs production. This is the first report of the presence of DTXs in cowpea plants colonized by the entomopathogenic fungus *M. robertsii*.

Not all fungal isolates tested here have been classified according to the Bischoff et al. [35] protocol, but based on the species names attributed to these isolates in the ARSEF catalog (Table 1), we note that the production of DTXs is not strictly correlated with *Metarhizium* species. For example: ARSEF 552 (*M. pingshaense*) and ARSEF 2575 (*M. robertsii*) are producers of DTXs *in vitro*, while isolates ARSEF 1448 (*M. pingshaense*) and ARSEF 724 (*M. robertsii*) are not. In the present study, *M. acridum* isolate ARSEF 324 did not produce detectable levels of DTXs after culture *in vitro* for 5 days; which is similar to the findings of Wang et al. [27] with this isolate *in vitro*. In contrast, Kershaw et al. [19] and Moon et al. [26] reported low levels of DTXs A and E production *in vitro* by isolate ARSEF 324 with longer incubation periods and higher temperatures.

Comparisons of DTXs production *in vitro* with virulence to insects of the 20 *Metarhizium* spp. isolates did not indicate a close association of the two traits. The most virulent isolates for *T. molitor* were ARSEF 3643, ARSEF 1448 (both isolates caused 100% mortality 5 days after treatment with 1×10^7^ conidia mL^−1^). Interestingly, ARSEF 3643 was the best DTX producer *in vitro*, while there were no detectable levels of DTXs produced by ARSEF 1448 in liquid culture. The same occurred with *G. mellonella*, i.e., of the 5 most virulent isolates (e.g., ARSEFs 724, 760, 1885, 2575, 3641, and 3643) two did not produce DTXs *in vitro* (ARSEF 724 and ARSEF 760), one was a very weak producer *in vitro* (ARSEF 1885), and three were good DTXs producers *in vitro* (ARSEF 2575, ARSEF 3641, and especially ARSEF 3643). Another trait that might relate to virulence is the first time (date) that DTXs were detectable in culture supernatants. For most DTXs producing isolates, these compounds were detected in liquid culture on day 3 of fungal growth; however, ARSEF 759 had detectable levels of DTX E at day 2 in culture (Figure 2). ARSEF 759 did not demonstrate higher potency or a shorter lethal time in comparison to fungal isolates that only showed detectable levels at days 3 or 4 in culture (e.g., ARSEF 2575 and ARSEF 1885). These observations suggest that the presence or the absence of DTXs A, B, and E in *in vitro* culture supernatants had little or no correlation with percent mortality or speed of insect kill.

Arthropod pathogens such as *B. bassiana* [31], [36], [37]; *Lecanicillium lecanii* ( =  *Verticillium lecanii*) [37], [38]; *Isaria farinosa* ( =  *Paecilomyces farinosus*) [39]; and *M. robertsii* [23] have been reported as endophytes. According to O'Brien [40], *M. acridum*, an acridid specialist, is not rhizosphere-competent; and Pava-Ripoll et al. [29] reported that germination of this fungal species in plant root exudates was significantly lower than with *M. robertsii* ( =  *M. anisopliae*). On the other hand, as reported in the current study, *M. acridum* colonized endophytically either cowpea or cucumber when surface sterilized seeds were inoculated with conidia in the laboratory. It currently is not known if spraying leaves of plants with this fungus will permit endophytic establishment in leaves, stems and roots.

The entomopathogenic fungus *B. bassiana* is an endophyte in naturally colonized plants [36], and also has been isolated after artificial inoculation in many important agricultural crops such as bananas, bean, coffee, corn, cotton, tomato and wheat [37]. Bing and Lewis [41] reported that tunneling in corn plants by *Ostrina nubilalis* larvae, the European corn borer, was reduced when plants were endophytically colonized by *B. bassiana*. Although the overwhelming majority of publications on the use of arthropod-pathogenic fungi against insects discuss the reduction of insect damage through insect death due to direct fungal infection by conidia, Vega et al. [36] suggested that this suppression of insect damage in response to *B. bassiana* plant colonization [41] may be the result of feeding deterrence or antibiosis. Such deterrence by some fungi is related to their production of metabolites. More recently, Gurulingappa et al. [37] studied the effect of endophytes (*B. bassiana*, *L. lecanii* and *Aspergillus parasitucus*) on the reproduction and growth of *Aphis gossypii* and *Chortoicetes terminifera*. They reported that endophytes significantly reduced aphid reproduction and locust growth rate, but no direct mortality was observed. Amiri et al. [16] reported residual and antifeedant activities of DTXs A, B, and E when leaf discs of Chinese cabbage were immersed in these toxins and submitted to larvae of crucifer pests *P. xylostella* and *P. cochleariae*; as a result, leaf area ingested by these larvae was greater for untreated leaves than DTXs-treated leaves in doses higher than 3 µg/g [16]. According to the study [16] with crucifer pest larvae, the amount of DTXs detected in cowpeas in the current study should be slightly toxic. However, DTXs amounts in plants older than those that we studied probably would vary, and DTXs susceptibility of other insect species also are likely to vary. The mechanisms involved in feeding suppression of insects by contact and/or ingestion of DTXs remains unclear.

*Metarhizium* spp. have been indicated as mediators of interactions among plants, insects and soil: e.g., Behie et al. [22] showed that plants can receive significant amounts of nitrogen from *Metarhizium*-infected soil insects. Sasan and Bidochka [23] reported that endophytic establishment of *M. robertsii* in roots induced growth of plant roots and root hairs. The ability of some *Metarhizium* isolates to produce DTXs within plants, as reported here, suggests another potentially important benefit to plants from endophytic association with these fungi.

DTXs production by AP fungi in plants depends not only on the fungal isolate but also on the plant species. Our results showed that even when colonized with *M. robertsii* ARSEF 2575 (an isolate that produces DTXs *in vitro* and also in cowpeas), cucumber extracts did not have detectable levels of DTXs. A plant pathogen *Alternaria brassicae*, the causative agent of *Alternaria* blackspot, is known to produce DTX B that is used to facilitate plant colonization. DTX B is a selective toxin, in that only plant cultivars susceptible to the toxin are damaged by the fungus [5], [42]. Resistant plants have enzymes that detoxify DTX B [42]. The current study did not investigate whether cucumber plants hydrolyzed DTX or if this host plant did not support DTX production.

Further studies on the effects of *per os* DTXs exposure in vertebrate organisms are needed to support the use of entomopathogenic fungi inoculated in crop seeds to control insect pests. In an instance where there is some hesitancy by regulating agencies about allowing DTXs in a food product, a non-DTXs producing isolate of *Metarhizium* could be selected for use in biological control on that crop to avoid such DTXs production, or plant cultivars that detoxify DTXs could be selected.

*In planta* production of secondary metabolites by endophytic *Metarhizium* may be an exploitable feature of this fungus in its use against agricultural arthropod pests. The production of DTXs in *M. robertsii*-colonized plants reported here clearly indicates that further investigation is warranted on the antifeedant or repellent properties of fungal metabolites expressed *in planta*.

## Supporting Information

Table S1 **Destruxin production (mg/L) by 12** ***Metarhizium*** **spp. isolates** ***in vitro*** **.** Destruxin production is represented by mean values ± standard error after 5 days in submerged shaken cultures.
(DOC)

## References

[1] Bittencourt VREP, Massard CL, Lima AF (1992) Uso do fungo *Metarhizium anisopliae* (Metschnikoff, 1879) Sorokin, 1883, no controle do carrapato *Boophilus microplus* (Canestrini, 1887). Arquivo da Universidade Rural do Rio de Janeiro 15: 197–202.

[2] Fernandes EKK, Bittencourt VREP (2008) Entomopathogenic fungi against South American tick species. Exp Appl Acarol 46: 71–93. 18563593 10.1007/s10493-008-9161-y

[3] Roberts DW, St. Leger RJ (2004) *Metarhizium* spp., cosmopolitan insect pathogenic fungi: mycological aspects. Adv Appl Microbiol 54: 1–70. 15251275 10.1016/S0065-2164(04)54001-7

[4] Samish M, Ginsberg H, Glaser I (2004) Biological control of ticks. Parasitology 129: S389–S403. 15938520 10.1017/s0031182004005219

[5] Pedras MSC, Zaharia LI, Ward DE (2002) The destruxins: synthesis, biosynthesis, biotransformation, and biological activity. Phytochemistry 59: 579–596. 11867090 10.1016/s0031-9422(02)00016-x

[6] Samuels RI, Charnley AK, Reynolds SE (1988) The role of destruxins in the pathogenicity of 3 strains of *Metarhizium anisopliae* for the tobacco hornworm *Manduca sexta* . Mycopathologia 104: 51–58.

[7] Samuels RI, Reynolds SE, Charnley AK (1988) Calcium channel activation of insect muscle by destruxins, insecticidal compounds produced by the entomopathogenic fungus *Metarhizium anisopliae* . Comp Biochem Physiol 90C: 403–412.

[8] Quiot JM, Vey A, Vago C (1985) Effects of mycotoxins on invertebrate cells *in vitro* . Adv Cell Cult 4: 199–212.

[9] James PJ, Kershaw MJ, Reynolds SE, Charnley AK (1993) Inhibition of desert locust (*Schistocera gregaria*) Malpighian tubule fluid secretion by destruxins, cyclic peptide toxins from the insect pathogenic fungus *Metarhizium anisopliae* . J Insect Physiol 39: 797–804.

[10] Muroi M, Shiragami N, Takatsuki A (1994) Destruxin B, a specific and readily reversible inhibitor of vacuolar-type H+-translocating ATPase. Biochem Biophys Res Commun 205: 1358–1365. 7802670 10.1006/bbrc.1994.2815

[11] Cerenius L, Thornqvist P, Vey A, Johansson MW, Soderhall K (1990) The effect of the fungal toxin destruxin E on isolated crayfish haemocytes. J Insect Physiol 36: 785–789.

[12] Han P, Jin F, Dong X, Fan J, Qiu B, Ren S (2013) Transcript and protein analysis of the destruxin A-induced response in larvae of *Plutella xylostella* . Plos One 8: e60771–e60781. 23585848 10.1371/journal.pone.0060771 PMC3621956

[13] Huxham IM, Lackie AM, McCorkindale NJ (1989) Inhibitory effects of cyclodepsipeptides, destruxins, from the fungus *Metarhizium anisopliae*, on cellular immunity in insects. J Insect Physiol 35: 97–105.

[14] Vey A, Matha V, Dumas C (2002) Effects of the peptide mycotoxin destruxin E on insect haemocytes and on dynamics and efficiency of the multicellular immune reaction. J Invertbr Pathol 80: 177–187. 10.1016/s0022-2011(02)00104-0 12384084

[15] Vilcinskas A, Matha V, Götz P (1997) Inhibition of phagocytic activity of plasmatocytes isolated from *Galleria mellonella* by entomogenous fungi and their secondary metabolites. J Insect Physiol 43: 475–483. 10.1016/s0022-1910(97)00066-8 12770487

[16] Amiri B, Ibrahim L, Butt TM (1999) Antifeedant properties of destruxins and their potential use with the entomogenous fungus *Metarhizium anisopliae* for improved control of crucifer pests. Biocontrol Sci Technol 9: 487–498.

[17] Thomsen L, Eilenberg J, Esbjerg P (1996) Effects of destruxins on *Pieris brassicae* and *Agrotis segetum* In: Smits PH, editor. Insect pathogens and insect parasitic nematodes. IOBC Bulletin 19: : 190–195.

[18] Donzelli BGG, Krasnoff SB, Sun-Moon Y, Churchill ACL, Gibson DM (2012) Genetic basis of destruxin production in the entomopathogen *Metarhizium robertsii* . Curr Genet 58: 105–116. 22367459 10.1007/s00294-012-0368-4

[19] Kershaw MJ, Moorhouse ER, Bateman R, Reynolds SE, Charnley AK (1999) The role of destruxins in the pathogenicity of *Metarhizium anisopliae* for three species of insect. J Invertebr Pathol 74: 213–223. 10534408 10.1006/jipa.1999.4884

[20] Suzuki A, Kawakami K, Tamura S (1971) Detection of destruxins in silkworm larvae infected with *Metarhizium anisopliae* . Agr Biol Chem 35: 1641–1643.

[21] Skrobek A, Shah FS, Butt TM (2008) Destruxin production by the entomogenous fungus *Metarhizium anisopliae* in insects and factors influencing their degradation. Biocontrol 53: 361–373.

[22] Behie SW, Zelisko PM, Bidochka MJ (2012) Endophytic insect-parasitic fungi translocate nitrogen directly from insects to plants. Science 336: 1576–1577. 22723421 10.1126/science.1222289

[23] Sasan RK, Bidochka M (2012) The insect-pathogenic fungus *Metarhizium robertsii* (Clavicipitaceae) is also an endophyte that stimulates plant root development. Am J Bot 99: 101–107. 22174335 10.3732/ajb.1100136

[24] St. Leger RJ (2008) Studies on adaptations of *Metarhizium anisopliae* to life in the soil. J Invertebr Pathol 98: 271–276. 18430436 10.1016/j.jip.2008.01.007

[25] Wyrebek M, Huber C, Sasan RK, Bidochka MJ (2011) Three sympatrically occurring species of *Metarhizium* show plant rhizosphere specificity. Microbiology 157: 2904–2911. 21778205 10.1099/mic.0.051102-0

[26] Moon Y-S, Donzelli BGG, Krasnoff SB, McLane H, Griggs MH, et al (2008) *Agrobacterium*-mediated disruption of a nonribosomal peptide synthetase gene in the invertebrate pathogen *Metarhizium anisopliae* reveals a peptide spore factor. App Environ Microbiol 74: 4366–4380. 10.1128/AEM.00285-08 PMC2493175 18502925

[27] Wang B, Kang Q, Lu Y, Bai L, Wang C (2012) Unveiling the biosynthetic puzzle of destruxins in *Metarhizium* species. Proc Natl Acad Sci U S A 109: 1287–1292. 22232661 10.1073/pnas.1115983109 PMC3268274

[28] Krasnoff SB, Sommers CH, Moon Y-S, Donzelli BGG, Vandenberg JD, et al (2006) Production of mutagenic metabolites by *Metarhizium anisopliae* . J Agric Food Chem 54: 7083–7088. 16968066 10.1021/jf061405r

[29] Pava-Ripoll M, Angelini C, Fang W, Wang S, Posada F, et al (2011) The rhizosphere-competent entomopathogen *Metarhizium anisopliae* expresses a specific subset of genes in plant root exudates. Microbiology 157: 47–55. 20947574 10.1099/mic.0.042200-0

[30] Rangel DNE, Fernandes EKK, Braga GUL, Roberts DW (2011) Visible light during mycelial growth and conidiation of *Metarhizium robertsii* produces conidia with increased stress tolerance. FEMS Microbiol Lett 315: 81–86. 21204917 10.1111/j.1574-6968.2010.02168.x

[31] Parsa S, Ortiz V, Vega FE (2013) Establishing fungal entomopathogens as endophytes: towards endophytic biological control. JoVE 74: e50360. 10.3791/50360 PMC3654456 23603853

[32] Tulloch M (1976) The genus *Metarhizium* . Trans Brit Mycol Soc 66: 407–411.

[33] Ayres M, Ayres JR M, Ayres DL, Santos AAS (2007) BioEstat 5.0 - Aplicações Estatísticas nas Áreas das Ciências Biológicas e Médicas. Sociedade Civil Mamirauá, Tefé, Brazil, 380 p.

[34] Sampaio IBM (2002) Estatística Aplicada à Experimentação Animal. Belo Horizonte, Brazil: FEPMVZ-Editora. 265 p.

[35] Bischoff JF, Rehner SA, Humber RA (2009) A multilocus phylogeny of the *Metarhizium anisopliae* lineage. Mycologia 101: 512–530. 19623931 10.3852/07-202

[36] Vega FE, Posada F, Aime MC, Pava-Ripoll M, Infante F, et al (2008) Entomopathogenic fungal endophytes. Bio Control 44: 72–82.

[37] Gurulingappa P, Sword GA, Murdoch G, McGee PA (2010) Colonization of crop plants by fungal entomopathogens and their effect on two insect pests when *in planta* . Bio Control 55: 34–41.

[38] Petrini O (1981) Endophytische pilze in *Epiphytischen araceae*, *Bromeliaceae* and *Orchidiaceae* . Sydowia 34: 135–148.

[39] Bills GF, Polishook JD (1991) Microfungi from *Carpinus caroliniana* . Can J Bot 69: 1477–1482.

[40] O'Brien TR (2008) *Metarhizium anisopliae*'s persistence as a saprophyte, genetic basis of adaptation and role as a plant symbiont. PhD Dissertation, University of Maryland. Available: http://drum.lib.umd.edu/handle/1903/8839. Accessed 2013 July 05.

[41] Bing LA, Lewis LC (1991) Suppression of *Ostrinia nubilalis* (Hubner)(Lepdoptera: Pyralidae) by endophytic *Beauveria bassiana* (Balsamo) Vuillemin. Environ Entomol 20: 1207–1211.

[42] Pedras MSC, Zaharia IL, Gai Y, Zhou Y, Ward DE (2001) *In planta* sequential hydroxylization and glycosylation of a fungal phytotoxin: avoiding cell death and overcoming the fungal invader. Proc Natl Acad Sci U S A 98: 747–752. 11149945 10.1073/pnas.021394998 PMC14659

