# Supplementary material for: Production of Destruxins from Metarhizium spp. Fungi in Artificial Medium and in Endophytically Colonized Cowpea Plants
Source: PLoS One. 2014 Aug 15;9(8):e104946. doi: 10.1371/journal.pone.0104946 (PMC4134251; doi:10.1371/journal.pone.0104946)
Supplement: Table S1 — Destruxin production (mg/L) by 12 Metarhizium spp. isolates in vitro . Destruxin production is represented by mean values ± standard error after 5 days in submerged shaken cultures. (DOC) [file pone.0104946.s001.doc]

| **Table S1: Destruxin production (mg/L) by 12 *Metarhizium* spp. isolates *in vitro.*** Destruxin production is represented by mean values ± standard error after 5 days in submerged shaken cultures. | | | |
| --- | --- | --- | --- |
| **Fungal Isolate** | **DTX E (mg/L)** | **DTX A (mg/L)** | **DTX B (mg/L)** |
| ARSEF 1885 | 0.94 ± 0.90 | 0.14 ± 0.03 | 0.66 ± 0.21 |
| ARSEF 1449 | 4.50 ± 1.09 | 1.20 ± 0.27 | 0.91 ± 0.35 |
| ARSEF 2211 | 16.16 ± 13.64 | 3.19 ± 2.61 | 2.61 ± 2.13 |
| ARSEF 3641 | 17.59 ± 2.44 | 3.50 ± 0.88 | 4.59 ± 1.52 |
| ARSEF 759 | 26.29 ± 9.72 | 3.01 ± 0.84 | 2.29 ± 0.88 |
| ARSEF 729 | 33.70 ± 17.77 | 3.73 ± 1.64 | 2.85 ± 1.94 |
| ARSEF 2575 | 50.20 ± 12.87 | 25.43 ± 6.74 | 56.42 ± 16.27 |
| ARSEF 929 | 58.74 ± 15.61 | 7.90 ± 0.97 | 6.31 ± 0.51 |
| ARSEF 148 | 60.89 ± 20.56 | 11.54 ± 3.37 | 10.47 ± 0.51 |
| ARSEF 552 | 87.46 ± 21.23 | 28.99 ± 3.91 | 19.96 ± 2.06 |
| ARSEF 2521 | 102.67 ± 27.68 | 23.97 ± 4.92 | 13.77 ± 2.95 |
| ARSEF 3643 | 188.21 ± 23.78 | 52.34 ± 2.20 | 34.65 ± 3.28 |
| Production of destruxins (DTXs) in supernatant of cultures was determined by quantitative HPLC analysis of the major components, viz., DTXs A, B and E. Cultures and assays were repeated 3 times. | | | |
